# Acknowledgment to the Reviewers of *EJIHPE* in 2022

**DOI:** 10.3390/ejihpe13010015

**Published:** 2023-01-13

**Authors:** EJIHPE Editorial Office

**Affiliations:** MDPI AG, St. Alban-Anlage 66, 4052 Basel, Switzerland

High-quality academic publishing is built on rigorous peer review. *EJIHPE* was able to uphold its high standards for published papers due to the outstanding efforts of our reviewers. Thanks to the efforts of our reviewers in 2022, the median time to first decision was 23 days and the median time to publication was 50 days. Regardless of whether the articles they examined were ultimately published, the editors would like to express their appreciation and thank the following reviewers for the time and dedication that they have shown *EJIHPE*:
A. R. M. Saifuddin EkramKaren MoodyAbbas EdalatKarla Dhungana SainjuAbbate Costanza ScaffidiKároly BozsonyiAbdelaziz Mohamed Mohamed MosaadKathleen Moritz RudasillAdela BadauKazuhiro P. IzawaAdriana LisKimberly KellyAgnieszka Zelek-MolikKirthana Kunikullaya UbrangalaAlberto DionigiKsenija BazdaricAlejandro Santos-LozanoKuan-Ying HsiehAlexander RobitzschKun-Hua LeeAlicja KamińskaKwok Kuen TsangAmanda SorensenLaura M. Guerrero PuertaAmelia DíazLea FerrariAna AdanLiliana CrăciunAna Álvarez MuelasLiliya PoskotinovaAna Belén MartínLindsey Conlin MaxwellAna Junça-SilvaLinfeng SongAnastasia KyvelidouLisa M. GallagherAndreas AltorferLjiljana TasicAndree HartantoLluís CodinaAndrés PandiellaLoucia DemetriouAndrzej J. PiotrowskiLucia MonacisAnna ShutalevaLuis VivancoAnne Beate ReinertsenMarc LochbaumAnnika Helgadóttir DavidsenMarcela Alina FărcașiuAntonia PelegrínMarek JasinskiAnzhelika VoroshilovaMargarida SaraivaArkadiusz StanulaMaría del Carmen Olmos-GómezArne DekkerMaría del Mar Molero JuradoArnulfo Hernan Nava-ZavalaMaría Dolores Díaz-NogueraArturo Barraza MacíasMaria Gratiela IanosAyyub A. PatelMaría Leticia Meseguer-SantamaríaBeata ŁubiankaMaria Lidia MasciaBeatriz Álvarez-EmbarbaMaria Rosa Suñer SolerBence KopperMaria TotanBernhard StraussMarija ZotovićBettina PikoMarios PittalisBiljana Van RijnMarisalva FáveroBordás Carlos SalaveraMariusz WysokinskiBruce E. WinstonMarkus GebhardtBryan T. FroehleMartina PavlíkováCarmen Corujo-VélezMatteo Cotta RamusinoCarmine FinelliMaurice MarsCarsten MüllerMelody AlmrothCastro May PortuguezMia StokmansCecilia Latorre CosculluelaMichael NorbergCesar A. SoutulloMichail KalogiannakisChao-Chen ChenMichał BronikowskiCharalambos GnardellisMohamed AbouzidCheng-Fang YenMohammad Amin KuhailChokri KooliMonika FraniaChristine Manlai KwanMonika Łopuszańska-DawidCiro EspositoMorena MuziClara Mockdece NevesMostafizur Rahman KomolClarice TangNabil GmadaConstantinos NicolaouNataša VardaCristiano ScandurraNathan MiczoCristina NunesNicola DöringCrystal I. BryceNicola MagnavitaDaniel T. L. ShekNicoleta Andreea NeacsuDaniela HaluzaNieves Barahona EstebanDariusz DrążkowskiNikola KomlenacDariusz KrokNimrod LevinDavid AparisiNina TumosaDelfín Ortega-SánchezOfra HalperinDiana KwokOlga BakadorovaDiane BurkeOliva Ángel De-JuanasDimitrios StamovlasisÒscar FloresDios Miguel Ángel QueirugaPanaoura AretiDominique DecouchantPaweł DębskiEduardo Augusto Werneck RibeiroPedro MeloEdvard JohanssonPedro Román-GravánEileen BrittPerman GochyyevElena Carrillo-AlvarezPeter FiegerElena DruicaPhilip HodgsonElena LyaksoPierre-Yves Collart-DutilleulElena NavarroPietro SarassoElio CastagnolaPing ZouElisabetta SagonePiotr GronekEloy López MenesesRafał GerymskiEric R. BravermanRaquel Vaquero-CristobalErnesto LodiRoberto Sánchez-CabreroEvelia Franco ÁlvarezRocco ServidioFaisal F. HakeemRodrigo NovaFederica CaffaroRodríguez Francisco Manuel MoralesFei LuoRoman FreunbergerFeliu Jordi ColomerRomualdas MalinauskasFiorenzo MoscatelliRyuichi OhtaFlorence SpitzenstetterSabrina DemarieFrancesco CampaSamuel P. LeónFrancisco Javier Riesco GonzálezSandro SerpaFrancisco José García-MoroSanto Di NuovoFrancisco PerezSatu ViertiöFrancisco Sánchez-AlberolaSaulius ŠukysGabriel González-ValeroSen-Chi YuGabriela DrocSerena BianchiGillian RaabShinyi Carol LinGiovanni MaccioniSilvana Loana De Oliveira-SousaGiulio ArcangeliSílvia Delgado OlabarriagaGlen BatesSilvio LorenzettiGonçalo DiasSimon F. RoyGregorio Gonzalez-AlcaideSimone SilvaGuoxiao SunSiti Fardaniah Abdul AzizGuy TraininSofia MarquesHéctor BurgosSophia AnastasiouHéctor RiveraSophie CarterHelen BarrieStamatoula TsikrikaHesam KamalipourStanislava IvanovaHiroshi FujiokaStefania ManconeHiu Chuen LokStefano RuggieriHoi Leong Xavier WongStormi Pulver WhiteHsin-An ChangSu LuIgor GrabovacSujit DasInes GuedesSusana SilvaInes TestoniSven BrembergInmaculada MateoSzabo Dan-AlexandruIván FernándezTadayoshi AsakaIván Herrera-PecoTakashi NaruseJ. Matt McCraryTatjana FischerJamil ExtremeraTeresa Bobes-BascaranJanice HegewaldTeresa Maria SgaramellaJavier Cifuentes-FauraTheodora PapamitsouJee-Hyun HwangTiija RintaJenny PangeTin-Chun LinJerry T. MitchellTommaso TrombettaJitendra SinghTse-Yang HuangJoanna MazurTussardi Ilaria ToccoJoão BritoUranchimeg TudevdagvaJohn HowardValerie A. MatareseJolanta ChmielowiecVassilios MakrakisJorge Sánchez InfanteVera StaraJosé Carmelo Adsuar SalaVincenzo Cristian FrancavillaJose I. BaileVsevolod KonstantinovJosé María Fernández-BataneroWalter R. SchummJosé-María León-RubioWaylon J. HastingsJoseph McIntyreYu KoyamaJudith Cavazos ArroyoYuichiro OtsukaJulie Aitken SchermerYuta UchiyamaJulio AlmenaraZacarías Sánchez MiláJulio C. De La Torre-MonteroZhaohui Su


